# Two novel species and two new records of *Distoseptispora* from freshwater habitats in China and Thailand

**DOI:** 10.3897/mycokeys.84.71905

**Published:** 2021-11-08

**Authors:** Hong-Wei Shen, Dan-Feng Bao, Kevin D. Hyde, Hong-Yan Su, Darbhe J. Bhat, Zong-Long Luo

**Affiliations:** 1 College of Agriculture and Biological Sciences, Dali University, Dali 671003, Yunnan, China; 2 Center of Excellence in Fungal Research, Mae Fah Luang University, Chiang Rai 57100, Thailand; 3 School of Science, Mae Fah Luang University, Chiang Rai 57100, Thailand; 4 Department of Entomology and Plant Pathology, Faculty of Agriculture, Chiang Mai University, Chiang Mai 50200, Thailand; 5 Innovative Institute of Plant Health, Zhongkai University of Agriculture and Engineering, Guangzhou 510225, China; 6 No. 128/1-J, Azad Housing Society, Curca, Goa Velha, 403108, India

**Keywords:** 2 new taxa, Distoseptisporales, freshwater fungi, morphology, phylogeny, taxonomy

## Abstract

During investigations into freshwater fungi from the Great Mekong Subregion, four *Distoseptispora* taxa were collected from China and Thailand. Based on morphological characteristics, and phylogenetic analyses of combined LSU, ITS, SSU, TEF1-α, and RPB2 sequence data, two new species *Distoseptisporabangkokensis* and *D.lancangjiangensis* are introduced, and two known species *D.clematidis* and *D.thysanolaenae* were first reported in freshwater habitat. Illustrations and descriptions of these taxa are provided, along with comparisons with extant taxa in the genus.

## Introduction

Distoseptisporaceae was introduced by Su et al. (2016) based on morphological and phylogenetic analyses, with *Distoseptispora* as type genus. Distoseptisporaceae is placed in Distoseptisporales, which was introduced by Luo et al. (2019), and currently comprises two families, Aquapteridosporaceae and Distoseptisporaceae (Luo et al. 2019; Wijayawardene et al. 2020; Hyde et al. 2021). Species of both families are commonly reported from freshwater habitats (Yang et al. 2015, 2018; Su et al. 2016; Li et al. 2021; Hyde et al. 2016a, 2019, 2020; Luo et al. 2018, 2019; Song et al. 2020; Dong et al. 2021).

*Distoseptispora* as a single genus in Distoseptisporaceae was introduced by Su et al. (2016) with *D.fluminicola* as the type species. The genus is characterized by monoblastic, cylindrical, conidiogenous cells, with percurrent proliferation, acrogenous, solitary, brown or yellowish/reddish brown, olivaceous, distoseptate or euseptate, cylindrical, obclavate, rostrate conidia, truncate base, with rounded apices, basal cell with a cross wall and basal scar. This genus is not known for its sexual morph (Su et al. 2016; Yang et al. 2018; Hyde et al. 2019, 2020; Luo et al. 2019; Sun et al. 2020). Currently, 32 species are accepted in the genus of which 13 from terrestrial habitats and 19 were reported from freshwater environments (Su et al. 2016; Hyde et al. 2016a, 2019, 2020; Xia et al. 2017; Yang et al. 2018; Luo et al. 2018, 2019; Monkai et al. 2020; Song et al. 2020; Sun et al. 2020; Li et al. 2021; Index Fungorum 2021http://www.indexfungorum.org).

During our ongoing study of freshwater fungi along the north-south gradient in the Asian/Australian region (Hyde et al. 2016b), we collected four species in the genus. Two new species, *Distoseptisporabangkokensis* and *D.lancangjiangensis*, are introduced in this study, *D.clematidis* and *D.thysanolaenae* are newly recorded from freshwater habitats for the first time in China. Morphological descriptions and illustrations of the species and an updated multi-gene phylogenetic tree are provided to reveal their taxonomic position among the species in the Distoseptisporales, and also provided the comparison of morphological characteristics, habitats and hosts information of species newly added to *Distoseptispora* after Monkai et al. (2020) (Table 2).

## Materials and methods

### Isolation and morphology

Specimens of submerged decaying wood were collected from Dulongjiang, Nanpanjiang, Lancangjiang and Chao Phraya River in China and Thailand respectively. Multiple samples will be collected at each collection site at different times, allowing more strains to be obtained for each species. Methods of morphological observation and isolation follow Luo et al. (2018) and Senanayake et al. (2020). IFW (Tarosoft(R) Image Frame Work) was used for measurement of photomicrograph, and Adobe Photoshop CS5 software was used to process images for making photo-plates (Adobe Systems Inc., USA). Single spore isolation was performed according to the following steps: The conidia suspension from specimens, absorbed with a sterilized pipette, was placed on potato dextrose agar (PDA) and incubated at room temperature overnight. Germinated conidia were transferred to new PDA/MEA (Beijing land bridge technology CO., LTD., China) plates and incubated in an incubator at room temperature (25 °C). Specimens were deposited in the Kunming Institute of Botany, Academia Sinica herbarium (KUN-HKAS), and Mae Fah Luang University herbarium (MFLU). Cultures were deposited in the Dali University Culture Collection (DLUCC), China General Microbiological Culture Collection Center (CGMCC), and Mae Fah Luang University Culture Collection (MFLUCC). Facesoffungi number was obtained as described in Jayasiri et al. (2015) and Index Fungorum number was also registered (http://www.indexfungorum.org/Names/Names.asp). In this study, multiple samples were collected for each sample site and related environment, but unfortunately, there were still no more strains for the two new species in the paper.

### DNA extraction, PCR amplification, and sequencing

DNA extraction, PCR amplification, sequencing and phylogenetic analysis follow Dissayanake et al. (2020) with the following modifications. Fungal mycelia (200–500 mg) were scraped from grown on PDA/MEA plates using sterile scalpel, transferred to microcentrifuge tube with sterilized needles, and then grind with liquid nitrogen or quartz sand to break the cells. DNA was extracted using the Trelief^TM^ Plant Genomic DNA Kit (TSP101) according to the manufacturer’s instructions.

Five gene regions, LSU, ITS, SSU, TEF1-α, and RPB2 were amplified using LR0R/LR5, ITS5/ITS4, NS1/NS4, 983F/EF1-2218R, and RPB2-5F/RPB2-7cR (Vilgalys and Hester 1990; White et al. 1990; Liu et al. 1999) primer pairs respectively. Primer sequences are available at the WASABI database at the AFTOL website (aftol.org). The PCR mixture contained 12.5 μL of 2 × Power Taq PCR Master Mix (a premix and ready to use solution, including 0.1 Units/μL Taq DNA Polymerase, 500μm dNTP Mixture each (dATP, dCTP, dGTP, dTTP), 20 mm Tris-HCl pH 8.3, 100 Mm KCl, 3 mM MgCl_2_, stabilizer and enhancer), 1 μL of each primer including forwarding primer and reverse primer (10 μm), 1 μL template DNA extract and 9.5 μL deionized water (Luo et al. 2018). The PCR cycling conditions of LSU, ITS, SSU and TEF1-α were as follows: 94 °C for 3 min, followed by 35 cycles of denaturation at 94 °C for 30s, annealing at 55 °C for 50s, elongation at 72 °C for 1 min, and a final extension at 72 °C for 10 min. The PCR thermal cycle of RPB2 has a total of 40 cycles, and the conditions are as follows: initially denature at 95 °C for 5 min, and then enter 40 cycles: denaturation at 95 °C for 1 min, annealing at 52 °C for 2 min, extension at 72 °C for 90s, and finally at 72 °C for 10 min. PCR products were then purified using minicolumns, purification resin, and buffer according to the manufacturer’s protocols (Amersham product code: 27–9602–01). The sequences were carried out at Beijing Tsingke Biotechnology Co., Ltd. (Beijing, P.R. China).

### Phylogenetic analysis

Preliminary identification of genes obtained from fresh strains by GenBank database. The LSU, ITS, SSU, TEF1-α, and RPB2 used for phylogenetic analysis are selected based on the preliminary identification results and the related publications (Yang et al. 2018; Monkai et al. 2020). The sequences were aligned using MAFFT online service: Multiple alignment program for amino acid or nucleotide sequences MAFFT version 7 (Katoh and Standley 2013: http://mafft.cbrc.jp/alignment/server/index.html), and edited manually in BioEdit v. 7.0 (Hall 1999). The sequence dataset was combined using SquenceMatrix v.1.7.8 (Vaidya et al. 2011). The alignment formats were change to PHYLIP and NEXUS formats by ALigment Transformation EnviRonment (ALTER) website (http://sing.ei.uvigo.es/ALTER/).

Maximum likelihood (ML) analysis was carried out using the RAxML-HPC2 on XSEDE (8.2.12) (Stamatakis 2006; Stamatakis et al. 2008) of CIPRES Science Gateway website (Miller et al. 2010: http://www.phylo.org/portal2) and the estimated proportion of invariant sites is (GTRGAMMA+I) model.

Bayesian analyses were performed in MrBayes 3.2.6 (Ronquist et al. 2012) and the best-fit model (LSU, ITS, SSU, TEF1-α, and RPB2 are all GTR+I+G) of sequences evolution was estimated via MrModeltest 2.2 (Guindon and Gascuel 2003; Nylander 2004; Darriba et al. 2012). The Markov Chain Monte Carlo (MCMC) sampling approach was used to calculate posterior probabilities (PP) (Rannala and Yang 1996). Bayesian analyses of six simultaneous Markov chains were run for 10000000 generations with trees sampled every 1000 generations.

Phylogenetic trees were visualized using FigTree v1.4.0 (Rambaut 2012: http://tree.bio.ed.ac.uk/software/figtree/), editing and typesetting using Adobe Illustrator (AI) (Adobe Systems Inc., the United States). The new sequences were submitted in GenBank and the strain information used in this paper is provided in Table 1. The alignments and phylogenetic trees were deposited in TreeBASE (http://www.treebase.org/, accession number: 28758).

**Table 1.** Strains used for phylogenetic analysis and their corresponding GenBank numbers. The type strain are in bold font.

Species	Source	GenBank accession number					Reference
		LSU	ITS	TEF1-α	RPB2	SSU	
***Aquapteridosporafusiformis***	**MFLUCC 18-1606**	**MK849798**	**MK828652**	**MN194056**	–	–	Luo et al. (2019)
***A.lignicola***	**MFLUCC 15-0377**	**KU221018**	–	–	–	–	Yang et al. (2015)
*Distoseptisporaadscendens*	HKUCC 10820	DQ408561	–	–	DQ435092	–	Shenoy et al. (2006)
***D.appendiculata***	**MFLUCC 18-0259**	**MN163023**	**MN163009**	**MN174866**	–	–	Luo et al.(2019)
***D.aquatica***	**MFLUCC 15-0374**	**KU376268**	**MF077552**	–	–	–	Su et al. (2016)
***D.bambusae***	**MFLUCC 20-0091**	**MT232718**	**MT232713**	**MT232880**	**MT232881**	**MT232716**	Sun et al. (2020)
*D.bambusae*	MFLUCC 14-0583	MT232717	MT232712	–	MT232882	–	Sun et al. (2020)
***D.bangkokensis***	**MFLUCC 18-0262**	**MZ518206**	**MZ518205**	–	–	**MZ518208**	**This study**
***D.cangshanensis***	**MFLUCC 16-0970**	**MG979761**	**MG979754**	**MG988419**	–	–	Luo et al. (2018)
***D.caricis***	**CBS 146041**	**MN567632**	**MN562124**	–	**MN556805**	–	Crous et al. (2019)
***D.clematidis***	**MFLUCC 17-2145**	**MT214617**	**MT310661**	–	**MT394721**	**MT226728**	Phukhamsakda et al. (2020)
*D.clematidis*	KUN-HKAS 112708	MW879523	MW723056	MW729784	–	MW774580	This study
***D.dehongensis***	**KUMCC 18-0090**	**MK079662**	**MK085061**	**MK087659**	–	–	Hyde et al. (2019)
***D.euseptata***	**MFUCC 20-0154**	**MW081544**	**MW081539**	–	**MW151860**	–	Li et al. (2021)
*D.euseptata*	DLUCC S2024	MW081545	MW081540	MW084994	MW084996	–	Li et al. (2021)
***D.fasciculata***	**KUMCC 19-0081**	**MW287775**	**MW286501**	**MW396656**	–	–	Dong et al. (2021)
***D.fluminicola***	**MFLUCC 15-0417**	**KU376270**	**MF077553**	–	–	–	Su et al. (2016)
***D.guttulata***	**MFLUCC 16-0183**	**MF077554**	**MF077543**	**MF135651**	–	**MF077532**	Yang et al. (2018)
***D.hydei***	**MFLUCC 20-0115**	**MT742830**	**MT734661**	–	**MT767128**	–	Monkai et al. (2020)
***D.lancangjiangensis***	**KUN-HKAS 112712**	**MW879522**	**MW723055**	–	**MW882260**	–	**This study**
*D.leonensis*	HKUCC 10822	DQ408566	–	–	DQ435089	–	Shenoy et al. (2006)
***D.lignicola***	**MFLUCC 18-0198**	**MK849797**	**MK828651**	–	–	**MK828318**	Luo et al. (2019)
***D.longispora***	**HFJAU 0705**	**MH555357**	**MH555359**	–	–	**MH555431**	Song et al. (2020)
***D.martinii***	**CGMCC 3.18651**	**KX033566**	**KU999975**	–	–	**KX033537**	Xia et al. (2017)
*D.multiseptata*	MFLUCC 16-1044	MF077555	MF077544	MF135652	MF135644	MF077533	Yang et al. (2018)
***D.multiseptata***	**MFLUCC 15-0609**	**KX710140**	**KX710145**	**MF135659**	–	**NG_065693**	Hyde et al. (2016)
***D.neorostrata***	**MFLUCC 18-0376**	**MN163017**	**MN163008**	–	–	–	Luo et al. (2019)
***D.obclavata***	**MFLUCC 18-0329**	**MN163010**	**MN163012**	–	–	–	Luo et al. (2019)
***D.obpyriformis***	**MFLUCC 17-01694**	**MG979764**	–	**MG988422**	**MG988415**	–	Luo et al. (2018)
*D.obpyriformis*	DLUCC 0867	MG979765	MG979757	MG988423	MG988416	–	Luo et al. (2018)
***D.palmarum***	**MFLUCC 18-1446**	**MK079663**	**MK085062**	**MK087660**	**MK087670**	**MK079661**	Hyde et al. (2019)
***D.phangngaensis***	**MFLUCC 16-0857**	**MF077556**	**MF077545**	**MF135653**	–	**MF077534**	Yang et al. (2018)
***D.rayongensis***	**MFLUCC 18-0415**	**MH457137**	**MH457172**	**MH463253**	**MH463255**	**MH457169**	Hyde et al. (2012)
***D.rostrata***	**MFLUCC 16-0969**	**MG979766**	**MG979758**	**MG988424**	**MG988417**	–	Luo et al. (2018)
***D.saprophytica***	**MFLUCC 18-1238**	**MW287780**	**MW286506**	**MW396651**	**MW504069**	–	Dong et al. (2021)
***D.songkhlaensis***	**MFLUCC 18-1234**	**MW287755**	**MW286482**	**MW396642**	–	–	Dong et al. (2021)
***D.suoluoensis***	**MFLUCC 17-0224**	**MF077557**	**MF077546**	**MF135654**	–	**MF077535**	Yang et al. (2018)
*D.suoluoensis*	MFLUCC 17-1305	MF077558	MF077547	–	–	MF077536	Yang et al. (2018)
***D.tectonae***	**MFLUCC 12-0291**	**KX751713**	**KX751711**	**KX751710**	**KX751708**	–	Hyde et al. (2016)
D.tectonae*^1^	MFLUCC 16-0946	MG979768	MG979760	MG988426	MG988418	–	Luo et al. (2018)
***D.tectonigena***	**MFLUCC 12-0292**	**KX751714**	**KX751712**	–	**KX751709**	–	Hyde et al. (2016)
***D.thailandica***	**MFLUCC 16-0270**	**MH260292**	**MH275060**	**MH412767**	–	**MH260334**	Tibpromma et al. (2018)
***D.thysanolaenae***	**KUN-HKAS 102247**	**MK064091**	**MK045851**	**MK086031**	–	–	Phookamsak et al. (2019)
*D.thysanolaenae*	KUN-HKAS 112710	MW879524	MW723057	MW729783	–	–	This study
***D.xishuangbannaensis***	**KUMCC 17-0290**	**MH260293**	**MH275061**	**MH412768**	**MH412754**	**MH260335**	Tibpromma et al. (2018)
***D.yunnanensis***	**MFLUCC 20-0153**	**MW081546**	**MW081541**	**MW084995**	**MW151861**	–	Li et al. (2021)
***Myrmecridiumaquaticum***	**MFLUCC 15-0366**	**MK849804**	–	–	–	**MK828323**	Luo et al. (2019)
*M.aquaticum*	S-1158	MK849803	MK828656	MN194061	MN124540	MK828322	Luo et al. (2019)
***M.banksiae***	**CBS 132536**	**JX069855**	**JX069871**	–	–	–	Crous et al. (2012)
***Pseudostanjehughesiaaquitropica***	**MFLUCC 16-0569**	**MF077559**	**MF077548**	**MF135655**	–	**MF077537**	Yang et al. (2018)
***P.lignicola***	**MFLUCC 15-0352**	**MK849787**	**MK828643**	**MN194047**	**MN124534**	–	Luo et al. (2019)
***Sporidesmiumdulongense***	**MFLUCC 17-0116**	**MH795817**	**MH795812**	**MH801191**	**MH801190**	–	Luo et al. (2019)
***S.lageniforme***	**DLUCC 0880**	**MK849782**	**MK828640**	**MN194044**	**MN124533**	–	Luo et al. (2019)
***S.pyriformatum***	**MFLUCC 15-0620**	**KX710141**	**KX710146**	**MF135662**	**MF135649**	–	Hyde et al. (2016)
*S.thailandense*	MFLUCC 15-0617	MF077561	MF077550	MF135657	–	–	Yang et al. (2018)
***S.thailandense***	**MFLUCC 15-0964**	**MF374370**	**MF374361**	**MF370957**	**MF370955**	–	Zhang et al. (2017)

*^1^ Ex-type strain of *Distoseptisporasubmersa*.

## Results

### Phylogenetic analysis

**Figure 1.**
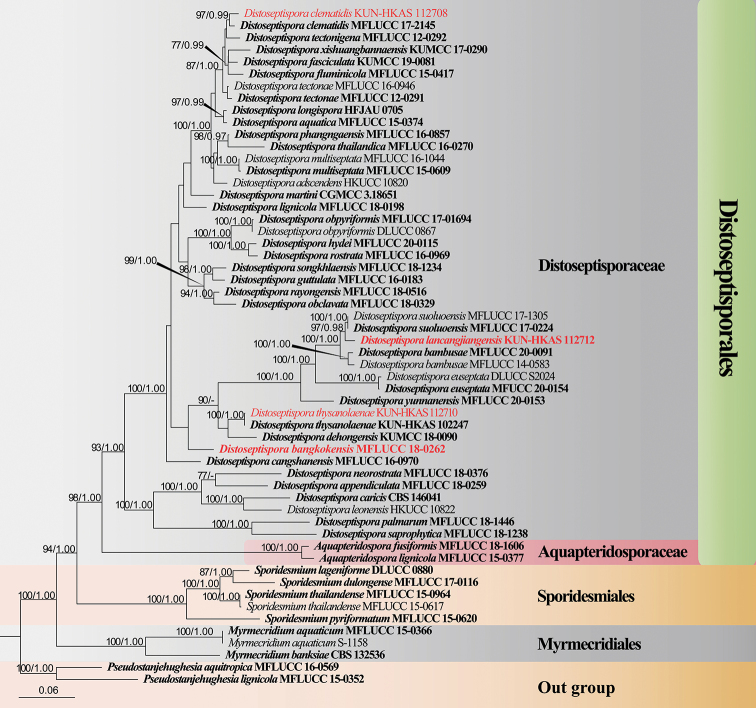
Maximum likelihood (ML) tree is based on combined of LSU, ITS, SSU, TEF1-α, and RPB2 sequence data. Bootstrap support values with an ML greater than 70% and Bayesian posterior probabilities (PP) greater than 0.97 given above the nodes, shown as “ML/PP”. The tree is rooted with *Pseudostanjehughesiaaquitropica* (MFLUCC 16-0569) and *P.lignicola* (MFLUCC 15-0352). New species are indicated in red and type strains are in bold.

The dataset composed of LSU (1–744 bp), ITS (745–1310 bp), TEF1-α (1311–2161 bp), RPB2 (2162–3178 bp), and SSU (3179–4199 bp) gene, comprising a total of 4199 characters (including gaps), including 56 taxa with *Pseudostanjehughesiaaquitropica* (MFLUCC 16-0569) and *P.lignicola* (MFLUCC 15-0352) as the outgroup taxa (Figure 1). The ML and BI phylogenetic analyses produced similar topology. The combined dataset analysis of RAxML generates a best-scoring tree (Figure 1), with the final ML optimization likelihood value of -30393.557997. The aligned matrix had 1624 distinct alignment patterns, with 36.44% completely undetermined characters or gaps. The base frequency and rate are as follows: A = 0.243915, C = 0.259360, G = 0.279029, T = 0.217696; rate AC = 1.166355, AG = 2.813539, AT = 1.110401, CG = 0.796371, CT = 5.621229, GT = 1.000000; gamma distribution shape: α = 0.221933. Bootstrap support values with a maximum likelihood (ML) greater than 70%, and Bayesian posterior probabilities (PP) greater than 0.97 are given above the nodes.

The phylogenetic tree shows that the new species *Distoseptisporabangkokensis* (MFLUCC 18-0262) was placed as a sister taxon to *D.bambusae* (MFLUCC 14-0583 and MFLUCC 20-0091), *D.dehongensis* (KUMCC 18-0090), *D.euseptata* (MFUCC 20-0154 and DLUCC S2024), *D.lancangjiangensis* (KUN-HKAS 112712), *D.suoluoensis* (MFLUCC 17-0224 and MFLUCC 17-1305), *D.thysanolaenae* (KUN-HKAS 102247 and KUN-HKAS 112710), and *D.yunnanensis* (MFLUCC 20-0153) with low bootstrap support with low bootstrap support (Figure 1), whereas *D.lancangjiangensis* clustered with *D.suoluoensis* with 97%ML/0.98PP support. *Distoseptisporathysanolaenae* (KUN-HKAS 112710) and *D.clematidis* (KUN-HKAS 112708) clustered with the ex-type strain of *D.thysanolaenae* (KUN-HKAS 102247) and *D.clematidis* (MFLUCC 17-2145), respectively, with 100%ML/1.00PP and 97%ML/0.99PP bootstrap support.

**Table 2.** Comparison of morphological characteristic, habitats and hosts’ information of species added to *Distoseptispora* after Monkai et al. (2020) (for other species see Monkai et al. 2020).

Species	Conidiophore (μm)	Conidia (μm)	Conidia septation	Conidia characteristic	Habitat	Host	Reference
*Distoseptisporabangkokensis*	37–55 × 3–4	400–568 × 13–16	Multi-distoseptate	Elongate, obclavate, rostrate, dark olivaceous to dark brown	Freshwater	Unidentified submerged wood	This study
*D.lancangjiangensis*	30–41 × 5–6	83–220 × 12–14	16–41-distoseptate	Obclavate, cylindrical, elongated, straight or curved, brown to greenish-brown	Freshwater	Unidentified submerged wood	This study
*D.euseptata*	19–28 × 4–5	37–54 × 8–9	4–7-euseptate	Obpyriform to obclavate, straight or curved, olivaceous	Freshwater	Unidentified submerged wood	Li et al. 2021
*D.fasciculata*	12–16 × 5–6	46–200 × 10–16.5	10–40-distoseptate	Subcylindrical to obclavate, mostly curved, olivaceous when young, dark brown when mature	Freshwater	Unidentified submerged wood	Dong et al. 2021
*D.longispora*	17–37 × 6–10	189–297 × 16–23	31–56-distoseptate	Obclavate, elongated, straight or slightly curved, to yellowish brown	Freshwater	Unidentified submerged wood	Song et al. 2020
*D.saprophytica*	50–140 × 3.2–4.2	14.5–30 × 4.5–7.5	2–6-distoseptate	Subcylindrical to obclavate, straight or curved, olivaceous to brown	Freshwater	Unidentified submerged wood	Dong et al. 2021
*D.songkhlaensis*	70–90 × 4–5.5	44–125 × 9–14.5	9–16-distoseptate	Obclavate, straight or curved, olivaceous to brown	Freshwater	Unidentified submerged wood	Dong et al. 2021
*D.yunnanensis*	131–175 × 6–7	58–108 × 8–10	6–10-euseptate	Obclavate, rostrate, straight or slightly curved, mid olivaceous to brown	Freshwater	Unidentified submerged wood	Li et al. 2021

### Taxonomy

#### Distoseptispora bangkokensis

Taxon classificationFungiDistoseptisporalesDistoseptisporaceae

H.W. Shen, D.F. Bao, K.D. Hyde & Z.L. Luo sp. nov.

E08487C9-F52C-559D-B571-BFE767D78DD4

Index Fungorum Number No: IF558556

Facesoffungi Number No: FoF09993

Figure 2

##### Etymology.

Referring to the collecting location, Bangkok, Thailand.

##### Holotype.

MFLU 21-0110

##### Description.

*Saprobic* on submerged wood in freshwater stream. **Sexual morph**: Undetermined. **Asexual morph**: *Colonies* effuse, glistening, hairy, brown to dark brown. *Mycelium* partly superficial in the substratum, composed of hyaline to pale brown, septate, branched hyphae. *Conidiophores* 37–55 × 3–4 μm (x¯ = 46 × 3 μm, n = 15) macronematous, mononematous, solitary or in a small group of 2–4, cylindrical, straight or slightly flexuous, 3–8-septate, dark brown, paler at the apical part, rounded at the apex. *Conidiogenous cells* 6–8 × 3–4 μm (x¯ = 7 × 3 μm, n = 15), integrated, terminal, monoblastic, cylindrical, brown. *Conidia* 400–568 × 13–16 μm (x¯ = 484 × 15 μm, n = 20), 6–7 μm at the narrowest apical region, acrogenous, solitary, elongate, obclavate, rostrate, multi-distoseptate, tapering towards the apex, truncate at the base, rounded at apex, dark olivaceous to dark brown, straight or slightly curved, guttulate, thick-walled, smooth, conidia percurrent proliferation which forms another conidium at the apex.

**Figure 2.**
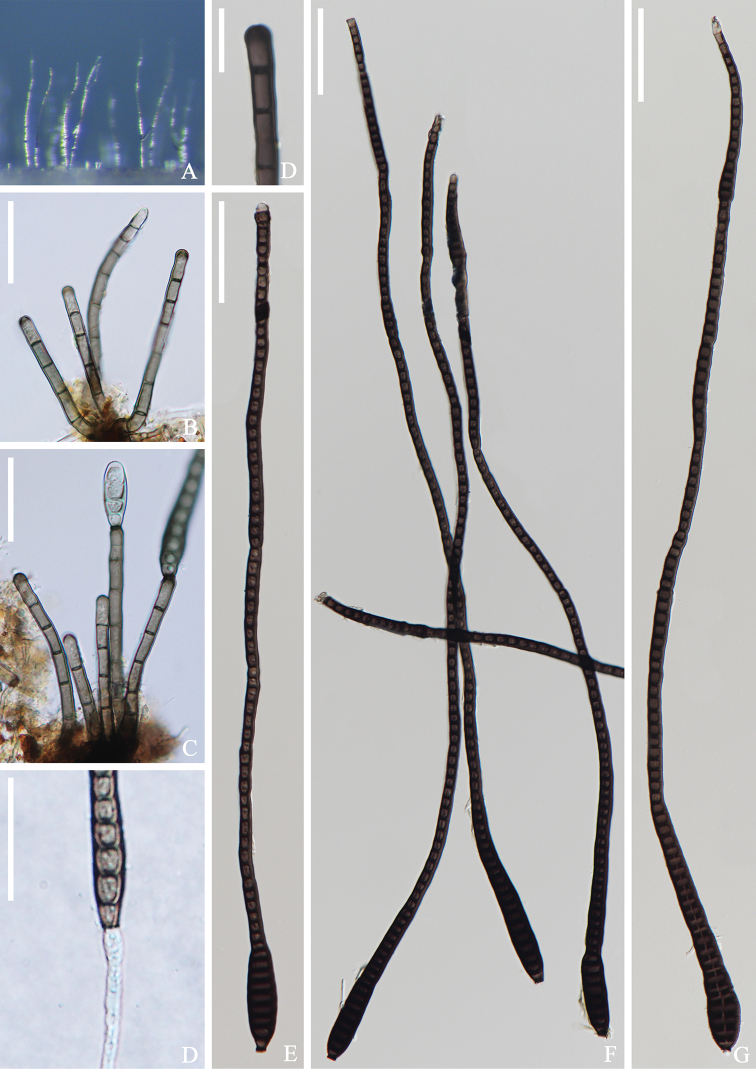
*Distoseptisporabangkokensis* (MFLU 21-0110, **holotype**) **A** colonies on the substratum **B** conidiophores **C** conidiophores with conidia **D** conidiogenous cell **E-G** conidia **H** germinating conidium Scale bars: 20 μm (**B, C, H**); 10 μm (**D**); 50 μm (**E-G**).

**Culture characteristics.** Conidia cultivated on PDA within 12h and germ tubes produced at the ends. Colonies on PDA, reaching 6 cm in 1 month at room temperature (25 °C). Mycelium loose, flocculent, smooth edge, brown to dark brown, dark brown on the reverse.

##### Material examined.

Thailand, Bangkok Province, Khwaeng Phra Khanong Nuea, 13°42'41"N; 100°36'03"E, on submerged decaying wood, 1 October 2017, Zonglong Luo, S–3083 (MFLU 21-0110, **holotype**), ex-type living culture (MFLUCC 18-0262).

##### Notes.

*Distoseptisporabangkokensis* is comparable to *D.cangshanensis* and *D.multiseptata* in having elongate, obclavate, or rostrate conidia (Su et al. 2016; Hyde et al. 2016a; Yang et al. 2018). However, *D.bangkokensis* has shorter and narrower conidiophores than those of *D.cangshanensis* (37–55 × 3–4 μm vs. 44–68 × 4–8 μm), but has longer conidia (400–568 μm vs. 58–166 μm); *D.multiseptata* (MFLU 17-0856) is similar to *D.bangkokensis* in conidial morphology, with conidia mostly 300–600 μm long (up to 700 μm) and significantly longer than those of the holotype (up to 380 μm long). However, Yang et al. (2018) did not give a detailed description of *D.multiseptata* (MFLU 17-0856). Phylogenetic analyses showed that *D.bangkokensis* clustered with *D.bambusae*, *D.dehongensis*, *D.euseptata*, *D.lancangjiangensis*, *D.suoluoensis*, *D.thysanolaenae*, and *D.yunnanensis* with low bootstrap support (26%ML/0.53PP, Figure 1). *Distoseptisporabangkokensis* is distoseptate conidia, and it is easily distinguished from *D.bambusae*, *D.euseptata*, *D.lancangjiangensis*, *D.suoluoensis*, and *D.yunnanensis*, which are euseptate. *Distoseptisporabangkokensis* is resemble to *D.dehongensis* and *D.thysanolaenae* in having obclavate, distoseptae conidia, but are distinguished by conidia characteristics, *D.bangkokensis* has elongate, obclavate, rostrate, multi-distoseptat, and longer conidia than *D.dehongensis* (400–568 × 13–16 μm vs. 17–30 × 7.5–10 μm) and *D.thysanolaenae* (400–568 × 13–16 μm vs. 30–70 × 5–8 μm), respectively.

#### Distoseptispora lancangjiangensis

Taxon classificationFungiDistoseptisporalesDistoseptisporaceae

H.W. Shen, H.Y. Su, K.D. Hyde & Z.L. Luo sp. nov.

BB47E43B-AC2E-525D-A336-5ED94C89D927

Index Fungorum Number No: IF558555

Facesoffungi Number No: FoF09994

Figure 3

##### Etymology.

Referring to the collecting location, Lancangjiang River in China.

##### Holotype.

KUN-HKAS 112712

##### Description.

*Saprobic* on submerged wood in freshwater River. **Sexual morph**: Undetermined. **Asexual morph**: *Colonies* effuse, hairy, glistening, brown to dark. *Mycelium* partly immersed in the substratum, composed of hyaline to pale brown, septate, branched hyphae. *Conidiophores* 144–204 × 5–6 μm (x¯ = 175 × 6 μm, n = 20) macronematous, mononematous, solitary, inflate at the base, cylindrical, straight or slightly flexuous, 6–11-septate, dark brown, hyaline and rounded at apex. *Conidiogenous cells* 12–24 × 4–5 μm (x¯ = 18 × 5 μm, n = 20) integrated, terminal, monoblastic, cylindrical, brown. *Conidia* 64–84 × 9–10 μm (x¯ = 74 × 10 μm, n = 20), acrogenous, solitary, narrowly obclavate or obspathulate, tracted at base, tapering towards apex, 3–10-euseptate, brown to dark brown, thin-walled, becoming paler or hyaline towards apex, guttulate, with a darkened scar at base, smooth-walled.

**Figure 3.**
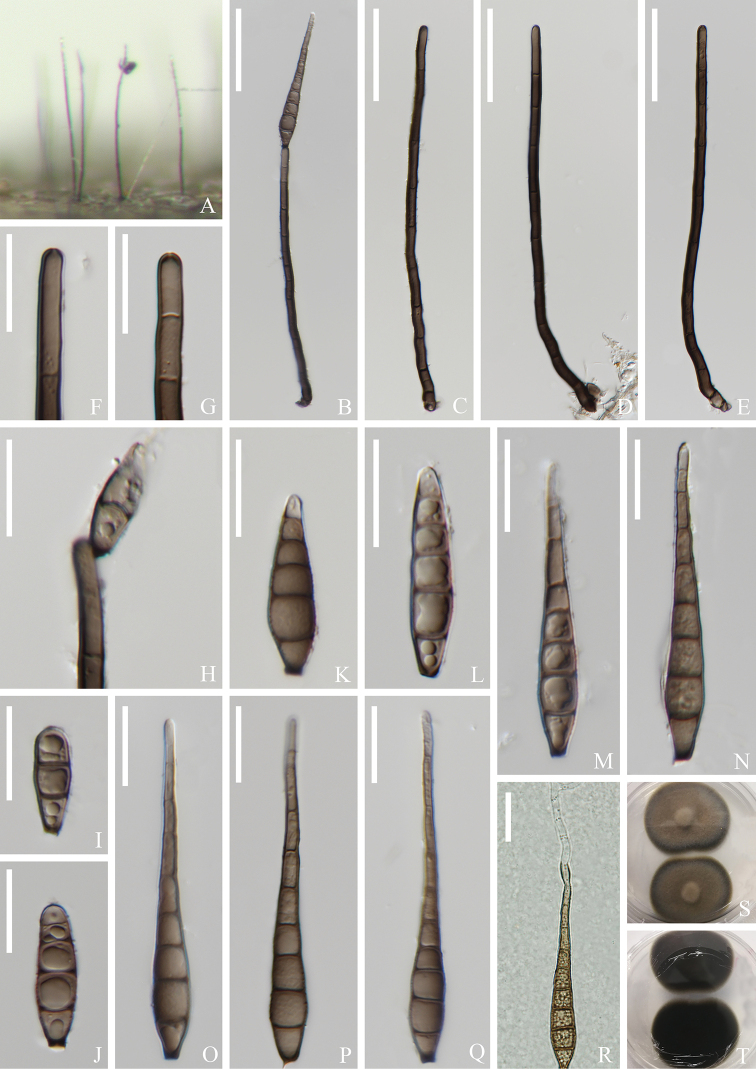
*Distoseptisporalancangjiangensis* (KUN-HKAS 112712, **holotype**) **A** colonies on the substratum **B** conidiophore and conidium **C-E** conidiophores **F, G** conidiogenous cells **H** conidiogenous cell with conidium **I-Q** conidia **R** germinating conidium **S, T** culture on PDA. Scale bars: 50 μm (**B-E**); 20 μm (**F-R**).

##### Culture characteristics.

Conidia cultivated on PDA within 12h and germ tubes produced at the apex. Colonies on PDA, reaching 4.5 cm in 1 month at room temperature (25 °C). Mycelium loose, flocculent, smooth edges, convex middle, pale brown to dark brown on the surface of PDA. Smooth, black on the reverse.

##### Material examined.

China, Yunnan Province, Dali City, Lancangjiang River, 22°36'36"N; 100°37'59"E, on submerged decaying wood, 20 July 2017, Qishan Zhou and Qingxiong Ruan S–1864 (KUN-HKAS 112712, ***holotype***; MFLU 21-0111, ***isotype***), ex-type living culture (DLUCC 1864 = CGMCC 3.20265).

##### Notes.

Phylogenetic analysis showed that *Distoseptisporalancangjiangensis* clustered as a sister taxon to *D.suoluoensis* with 97%ML/0.98PP support. *Distoseptisporalancangjiangensis* is similar to *D.suoluoensis* in having long conidiophores, monoblastic conidiogenous cells, and obclavate to rostrate, euseptate conidia. However, *D.suoluoensis* has yellowish-brown or dark olivaceous, verrucose conidia, while in *D.lancangjiangensis* conidia are brown to dark brown and smooth-walled. Moreover, *D.lancangjiangensis* has smaller conidia than those of *D.suoluoensis* (64–84 × 9–10 μm vs. 80–125 × 8–13 μm) (Yang et al. 2018). *Distoseptisporalancangjiangensis* and *D.bambusae* have similar conidial shapes, but *D.lancangjiangensis* having longer conidia (64–84 × 9–10 μm vs. 45–74 × 5.5–10 μm) and longer conidiophores (144–204 × 5–6 μm vs. 40–96 × 4–5.5 μm). Furthermore, *D.bambusae* has polyblastic or monoblastic conidiogenous cells and olivaceous or brown conidia, while *D.lancangjiangensis* only has monoblastic conidiogenous cells and brown to dark brown conidia (Sun et al. 2020).

#### Distoseptispora clematidis

Taxon classificationFungiDistoseptisporalesDistoseptisporaceae

Phukhams., M.V. de Bult & K.D. Hyde, in Phukhamsakda et al., Fungal Diversity 102: 168 (2020)

AC7F9A70-BD17-5567-BC09-C62CA24F0F50

Index Fungorum Number No: IF557301

Facesofungi Number No: FoF07261

Figure 4

##### Description.

*Saprobic* on submerged wood in freshwater River. **Sexual morph**: Undetermined. **Asexual morph**: *Colonies* on the substratum superficial, effuse, scattered, hairy, dark brown. *Mycelium* partly immersed in substrate, composed of branched, smooth, septate, brown to dark brown hyphae. *Conidiophores* 30–41 × 5–6 μm (x¯ = 36 × 6 μm, n = 15), macronematous, mononematous, single or in a small group, straight or slightly flexuous, unbranched, septate, erect, 2–4-septate, cylindrical, smooth, dark brown to brown. *Conidiogenous cells* 7–9 × 5–6 μm (x¯ = 8 × 5 μm, n = 15), monoblastic, integrated, determinate, terminal, cylindrical, pale brown to brown. *Conidia* 83–220 × 12–14 μm (x¯ = 151 × 13 μm, n = 20), acrogenous, solitary, obclavate, cylindrical, elongated, straight or curved, truncate at base, rounded at apex, 16–41-distoseptate, slightly constricted at some septa, smooth, brown to greenish-brown, thick-walled.

**Figure 4.**
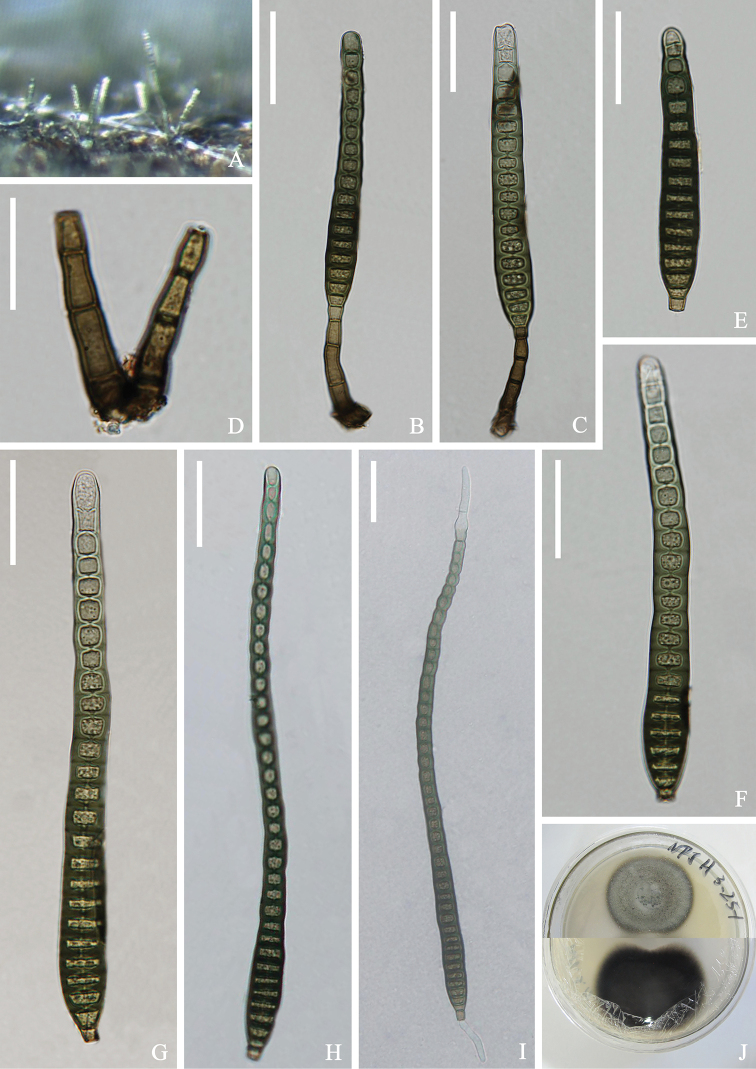
*Distoseptisporaclematidis* (KUN-HKAS 112708) **A** colonies on the substratum **B-C** conidiophores with conidia **D** conidiogenous cells **E-H** conidia **I** germinating conidium **J** culture on PDA Scale bars: 30 μm (**B, C, E-I**); 20 μm (**D**).

##### Culture characters.

Conidia cultivated on PDA within 12h and germ tubes produced at the ends. Colonies on PDA, attaining 4 cm after 1 month at room temperature (25 °C), gray at first, later becoming dark gray, loose, flocculent, smooth edge, dark brown on the reverse.

##### Material examined.

China, Yunnan Province, Kunming City, Yiliang County, Nanpanjiang River, 24°38'28"N; 103°09'38"E, on submerged decaying wood, 12 June 2018; Hongwei Shen and Xiu He, S–1797 (KUN-HKAS 112708), living culture (DLUCC 1797).

##### Notes.

Our new isolate clustered with the ex-type strain of *Distoseptisporaclematidis* (MFLU 17-1501) (Phukhamsakda et al. 2020) with 97%ML/0.99PP bootstrap support (Figure 1). *Distoseptisporaclematidis* (MFLU 17-1501) was collected on dead culms of *Thysanolaenamaxima* (Roxb. ex Hornem.) Honda in Yunnan Province, China. Based on morphological analysis, the size and shape of the conidia and conidiophores of our new isolate are similar to *D.clematidis*. Therefore, we identified our new isolate as *D.clematidis* and it is a new record from freshwater habitats in China.

#### Distoseptispora thysanolaenae

Taxon classificationFungiDistoseptisporalesDistoseptisporaceae

Goonas., Dayarathne, Phookamsak & K.D.Hyde, in Phookamsak et al., Fungal Diversity 95: 126 (2019)

68BD6629-9E92-5AC8-BE36-175569F80A7A

Index Fungorum Number No: IF555408

Facesoffungi Number No: FoF05011

Figure 5

##### Description.

*Saprobic* on submerged wood in freshwater River. **Sexual morph**: Undetermined. **Asexual morph**: *Colonies* on the substratum superficial, effuse, scattered, hairy, dark brown. *Mycelium* partly immersed, composed of branched, septate, smooth, brown to dark brown hyphae. *Conidiophores* 41–59 × 4–5 μm (x¯ = 50 × 5 μm, n = 20) macronematous, mononematous, unbranched, single, erect, straight or slightly curved, smooth, 3–6-septate, pale brown to brown. *Conidiogenous cells* monoblastic, integrated, determinate, terminal, dark brown, cylindrical. *Conidia* 46–87 × 9–12 μm (x¯ = 67 × 10 μm, n = 25) acrogenous, solitary, dry, smooth, obclavate, elongated, straight or slightly curved, truncate at base, tapering towards apex, 6–19-septate, dark grayish-brown to light yellow-green, thick-walled.

**Figure 5.**
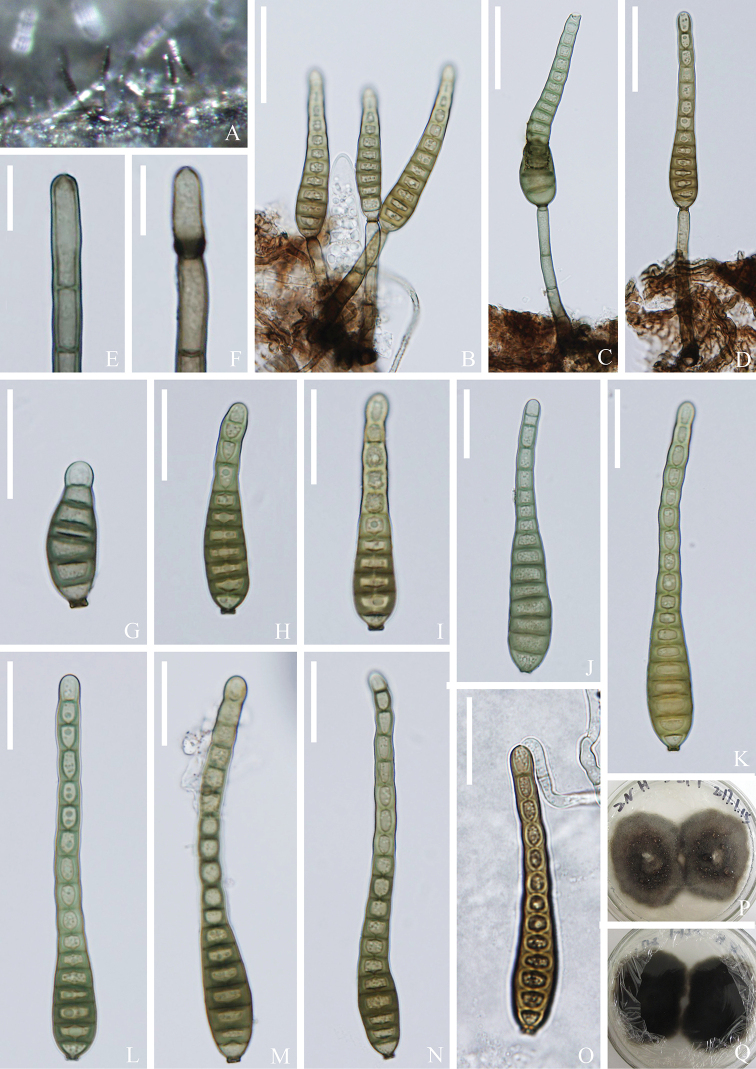
*Distoseptisporathysanolaenae* (KUN-HKAS 112710) **A** colonies on the substratum **B-D** conidiophores with conidia **E, F** conidiogenous cells **G-N** conidia **O** germinating conidium **P, Q** culture on PDA Scale bars: 30 μm (**B-D**); 10 μm (**E, F**); 20 μm (**G-O**).

##### Culture characteristics.

Conidia cultivated on PDA within 12 h and germ tubes produced at the apex. Colonies on PDA, reaching 6 cm after 6 weeks at room temperature (25 ℃). Mycelium loose, flocculent, neat edges, convex in middle, pale brown to dark brown. Black, smooth on the back.

##### Material examined.

China, Yunnan Province, Lushui City, Nujiang River, 26°23'12"N; 98°53'94"E, on submerged decaying wood, 3 May 2016, Zonglong Luo and Songming Tang, S-876 (KUN-HKAS 112710), living culture (DLUCC 876 = KUNCC 21-10710)

##### Notes.

Our new collection is identical to *Distoseptisporathysanolaenae* in characters of the conidiophores, conidiogenous cell, and conidia (Phookamsak et al. 2019). Furthermore, our new isolate phylogenetically clusters with the ex-type strain of *D.thysanolaenae* (KUN-HKAS 102247) with 100%ML/1.00PP support (Figure 1). *Distoseptisporathysanolaenae* was collected from terrestrial habitats in China, while, our new isolate was collected from freshwater habitat in China. Therefore, we identified our new collection as *D.thysanolaenae*, and it is new to freshwater habitats in China.

## Discussion

*Distoseptispora* has been reported from both freshwater and terrestrial habitats. Of these, species have been collected from freshwater environments (Su et al. 2016; Hyde et al. 2016a, 2019, 2020; Luo et al. 2018; Xia et al. 2017, 2019; Yang et al. 2018; Tibpromma et al. 2018; Crous et al. 2019; Phookamsak et al. 2019; Monkai et al. 2020; Phukhamsakda et al. 2020; Song et al. 2020; Sun et al. 2020; Li et al. 2021). To date, 18 species of *Distoseptispora* have been reported from Thailand, 14 species from China. In this study, we collected four *distoseptispora*-like taxa from rivers and streams in China and Thailand. Phylogenetic analysis showed that all four species were well-placed in *Distoseptispora* (Figure 1). Two new species and records are introduced based on morphological and phylogenetic analysis.

Species of *Distoseptispora* are highly diverse in morphology, especially the conidial shape. Conidia of most species are obclavate to cylindrical or rostrate (e.g. *D.aquatica*, *D.tectonae*, and *D.suoluoensis*), but a few are ellipsoid to subglobose (e.g. *D.martinii*), lanceolate (e.g. *D.guttulata* and *D.multiseptata*), and some species have conidia with a sheath at the apex (e.g. *D.appendiculata*) (Hyde et al. 2016a; Su et al. 2016; Xia et al. 2017; Yang et al. 2018; Luo et al. 2018, 2019). Some species also differ in the conidiogenous cells (*D.palmarum*, *D.dehongensis*, and *D.bambusae* are monoblastic or polyblastic, while the others are monoblastic) and conidial septate (*D.bambusae*, *D.euseptatensis*, *D.guttulata*, *D.lignicola*, *D.rayongensis*, *D.suoluoensis*, and *D.yunnanensis* are euseptate, while other species are distoseptate) (Yang et al. 2018; Hyde et al. 2019; Luo et al. 2019; Sun et al. 2020; Dong et al. 2021; Li et al. 2021).

Based on the key morphological characteristics, *viz.* conidiophores, conidiogenous cells, and conidia, Subramanian (1992) redisposed seven genera, *viz.*, *Sporidesmium*, *Polydesmus*, *Sporidesmiella*, *Stanjehughesia*, *Repetophragma*, *Penzigomyces*, and *Ellisembia* to accommodate several *Sporidesmium*-like taxa. Based on multi-gene phylogenetic analysis and morphology, Su et al. (2016) introduced a new *Sporidesmium*-like genus *Distoseptispora*. Some *Sporidesmium*-like taxa were introduced in different lineages and synonymized *Ellisembia* under *Sporidesmium*. Although *Distoseptispora* was only introduced from submerged wood in freshwater habitat in 2016 (Su et al. 2016), the genus has previously been reported from both freshwater and terrestrial habitats as species in other genera. For example, Cai et al. (2002), Ho et al. (2001, 2002) and Luo et al. (2004) reported *Distoseptispora* as other species (*Ellisembia*, *Sporidesmiella*, and *Sporidesmium*) from submerged wood in freshwater habitats, and Kodsueb et al. (2016), Mena-Portales et al. (2016) and Zhou et al (2001) reported from terrestrial habitats. However, none of these records had molecular data and it is impossible to consider the placement of these isolates. In these species *distoseptispora*/*sporidesmium*-like genera, it is therefore better to describe taxa based on molecular data.

Based on phylogenetic analysis, Xia et al. (2017) transferred *Acrodictysmartinii* to *Distoseptispora* as *Distoseptisporamartinii*. The species is characterized by solitary erect, unbranched conidiophores, monoblastic conidiogenous cells with percurrent extensions and subhyaline to pale brown and solitary, transversal ellipsoid, oblate or subglobose, muriform conidia, separated by septa, sometimes with pores in the septa and pale brown to brown. However, the current understanding of Distoseptisporaceae, *D.martinii* is significantly different from other *Distoseptispora* taxa; thus, needs to be verified in the future (Luo et al. 2018; Sun et al. 2020).

## Supplementary Material

XML Treatment for Distoseptispora bangkokensis

XML Treatment for Distoseptispora lancangjiangensis

XML Treatment for Distoseptispora clematidis

XML Treatment for Distoseptispora thysanolaenae
